# Species richness of Eurasian *Zephyrus* hairstreaks (Lepidoptera: Lycaenidae: Theclini) with implications on historical biogeography: An NDM/VNDM approach

**DOI:** 10.1371/journal.pone.0191049

**Published:** 2018-01-19

**Authors:** Hailing Zhuang, Masaya Yago, Josef Settele, Xiushan Li, Rei Ueshima, Nick V. Grishin, Min Wang

**Affiliations:** 1 Department of Entomology, College of Agriculture, South China Agricultural University, Guangzhou, Guangdong, China; 2 The University Museum, The University of Tokyo, Tokyo, Japan; 3 Department of Community Ecology, Helmholtz Centre for Environmental Research - UFZ, Halle, Saxony-Anhalt, Germany; 4 German Centre for Integrative Biodiversity Research (iDiv), Halle-Jena-Leipzig, Leipzig, Germany; 5 Institute of Biological Sciences, University of the Philippines Los Banos, Laguna, Philippines; 6 Institute of Biological Sciences, Graduate School of Science, The University of Tokyo, Tokyo, Japan; 7 Howard Hughes Medical Institute, University of Texas Southwestern Medical Center, Dallas, Texas, United States of America; University of Minnesota, UNITED STATES

## Abstract

**Aim:**

A database based on distributional records of Eurasian *Zephyrus* hairstreaks (Lepidoptera: Lycaenidae: Theclini) was compiled to analyse their areas of endemism (AoEs), species richness and distribution patterns, to explore their locations of past glacial refugia and dispersal routes.

**Methods:**

Over 2000 *Zephyrus* hairstreaks occurrences are analysed using the NDM/VNDM algorithm, for the recognition of AoEs. Species richness was calculated by using the option ‘Number of different classes’ to count the different classes of a variable presented in each 3.0°×3.0° grid cell, and GIS software was used to visualize distribution patterns of endemic species.

**Results:**

Centres of species richness of *Zephyrus* hairstreaks are situated in the eastern Qinghai-Tibet Plateau (EQTP), Hengduan Mountain Region (HDMR) and the Qinling Mountain Region (QLMR). Latitudinal gradients in species richness show normal distribution with the peak between 25° N and 35° N in the temperate zone, gradually decreasing towards the poles. Moreover, most parts of central and southern China, especially the area of QLMR-EQTP-HDMR, were identified as AoEs that may have played a significant role as refugia during Quaternary global cooling. There are four major distributional patterns of *Zephyrus* hairstreaks in Eurasia: Sino-Japanese, Sino-Himalayan, high-mountain and a combined distribution covering all three patterns.

**Conclusions:**

*Zephyrus* hairstreaks probably originated at least 23–24 Myr ago in E. Asia between 25° N to 35° N in the temperate zone. Cenozoic orogenies caused rapid speciation of this tribe and extrusion of the Indochina block resulted in vicariance between the Sino-Japanese and the Sino-Himalayan patterns. The four distribution patterns provided two possible dispersal directions: Sino-Japanese dispersal and Sino-Himalayan dispersal.

## Introduction

All living things on earth are not distributed randomly, but are restricted to a defined geographical area [1]. Two very important issues of biogeography are the identification of areas of endemism (AoEs) and centres of species richness. AoEs defined by at least two endemic taxa are generally treated as keys to link the distribution of organisms and historical geographical events [2, 3], and these areas are characterized with high species survival and speciation rates. Furthermore, these areas are widely regarded as refugia during the Last Glacial Maximum [4]. Consequently, they are usually treated as historical units used to infer the relationship between the distribution of organisms and historical factors [5–10]. Biogeographical boundaries, which are often affected by multiple factors (e.g. biological, physical, ecological, historical), have been of particular interest due to their impact on ecology, biogeography, evolution and conservation biology [11]. Biogeographical lines, the most famous of which is Wallace’s Line [12], are the boundaries defining the distribution of organisms. These lines reflect some consistency in the influence of ecological, physical, and geological factors, as well as historical events. For instance, the biogeographical line called the “Tanaka-Kaiyong line” which extends from northwest Yunnan through southeast Yunnan to Guangxi, China, and to northern Vietnam (about from 28° N, 98° E to 19° N, 108° E) was suggested by several researchers based on their floristic surveys [13–15].

Lepidoptera (moths and butterflies) form the second largest order in the class Insecta and are an ideal group for biogeographical studies due to their abundance and ubiquity [16–20]. However, the study on biogeography of Lepidoptera, especially at broad geographical scales, is not comprehensive enough due to lack of sufficiently detailed and available data in Eurasia. The *Zephyrus* hairstreaks (Lepidoptera: Lycaenidae: Theclini) are mainly distributed in Eurasia and are characterized by their brilliantly shining wings in a dazzling variety of metallic colours, such as metallic green, blue, purple, orange, silvery white and others. Therefore, they are often referred to as “living jewels”, enchanting naturalists, lepidopterists and amateurs alike [21]. This tribe was selected as our study objective for several reasons: Firstly, the outstanding taxonomic work, including 197 known species belonging to 53 genera of Theclini and their detailed distributional data reported prior to 2016, has been well synthesized [22–51]. Secondly, the description and revision of genera are beginning to stabilize, although there have been two new genera and some new species proposed since a relatively detailed survey of China and Indo-China in 2007, though there were several new species reported from northwest China and Indo-China [22, 28, 29, 31, 39, 41, 46–48, 51]. Thirdly, monophyly of this tribe was been discussed based on morphological features and ecological information [21, 22] and tested using molecular analyses (unpublished research by Zhuang et al., and Hsu et al.). However, their biogeographical relations are still poorly understood. By detecting the AoEs from the data, we can obtain new insights into the origins, diffusion and distribution of this group.

East Asia, located on the west side of the Pacific Ocean, is characterized by complex geological structures due to geographical events throughout history, such as the Indo-Asian tectonic collision which caused the formation of the Himalaya, uplifted the Qinghai-Tibetan plateau, and caused the rotation of the Shan-Malay Plate, and the glacial and interglacial periods during the Quaternary. The areas, affected by the collision and global cooling, are also biodiversity hotspots and are the focus of current research [23].

Our study examines the AoEs, centres of species richness of *Zephyrus* hairstreaks and their distribution patterns in Eurasia. By integrating the information from analyses based on our database, we aim to explain where the candidates of refugia of this tribe are and how the dispersal to the current distribution patterns can be reconstructed. This garnering information is useful for conservation applications.

## Materials and methods

### Species distribution data

We compiled a database including 2126 records of 197 known species belonging to 53 genera of Theclini. We included 1564 records of 189 Eurasian endemic species assigned to 47 genera to analyse species richness. Moreover, 1517 records of 142 endemic species from 42 genera (excluding species from only a single location) were used to identify areas of endemism (AoEs). Geographical distribution data and taxonomic information for each species were compiled and reviewed from data in literature published prior to 2016 [24–51]. Classification and nomenclature follow the literature of “The *Zephyrus* Hairstreaks of the World” by Koiwaya [22]. A list of species records and complete geographical information regarding the specimen collection sites and references can be found in the supporting information (Table in S1 Table).

### Description of the study area

An area covering latitudes -10° S to 80° N and longitudes from 40° E to 180° E, including most parts of the countries of Eurasia, was subdivided into quadrats measuring 2° × 2° and 3° × 3° Operative Geographical Units (OGU) without consideration of physiographical features and the uniformity of quadrats in the margin of the survey area that are only partly land [52]. This region covers most parts of the known distributional area of *Zephyrus* Hairstreaks in Eurasia.

### Analysis

Species richness is the most direct method to evaluate species diversity. A total of 1564 distributional records were coded as a shapefile that was then run in DIVA-GIS 7.5, using 3° × 3° grid cells. DIVA-GIS 7.5 is a free computer program for mapping and analyzing geographical distributions and biodiversity data [53–55]. Species richness was analyzed using the option “Number of different classes” to count the different classes of a variable present in each grid cell. A list of conditioned equal interval values was taken from a species richness map of the study area. Before analysis, a species accumulation curve at genus level has been tested, using our database, and the curve tends to go up and then it tends to slow down. It reflects a relatively adequate sampling.

NDM/VNDM 3, two sister programs written by Goloboff [56], implement the methods described in Szumik *et al*., and Szumik & Goloboff [57, 58] to demarcate AoEs. These programs, based on an optimality criterion, take into account the spatial component of endemism for identifying AoEs. Parameters were selected in accordance with Prado *et al*. [59]. Scores above 2.0 were saved for genus-level analysis and above 3.0 for species-level analysis, and set the upper minimum score above 0.3 (except for *Hayashikeia florianii* (0.289)). The consensus AoEs were computed using a cut-off of 50% similarity at different taxonomic levels and the flexible consensus was selected [56, 57]. Endemicity analysis (EA) was selected to identify areas of endemism and executed by NDM, because EA has been recognized as a best method that supported areas of endemism [60]. Analytical procedures are filed in http://dx.doi.org/10.17504/protocols.io.[dx.doi.org/10.17504/protocols.io.jr2cm8e].

AoEs were constructed from 2.0°×2.0° and 3.0°×3.0° cell sizes at each taxonomic level (genus and species). The two sizes of cells were optimized in consideration of the advantages and disadvantages of each particular size [59]. Different taxonomic levels used in the analysis of endemicity can increase the reliability [61, 62].

## Results

### Species richness of the tribe Theclini

**Fig 1:**
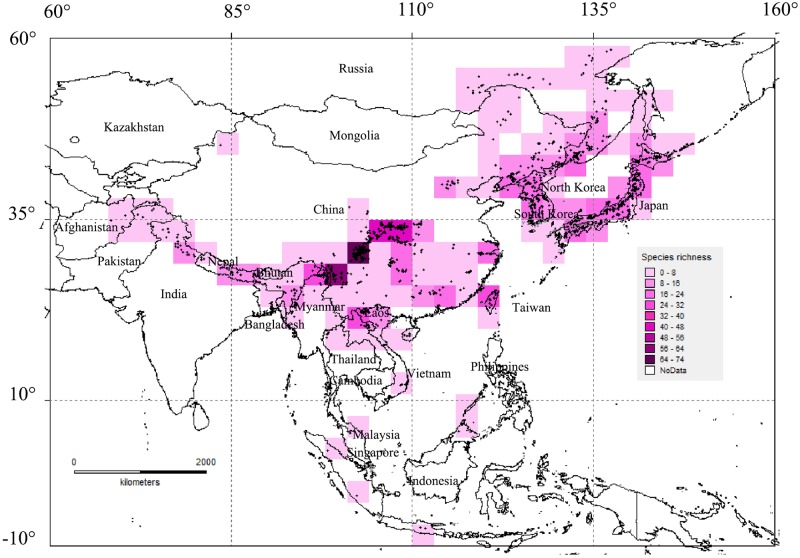
Species richness of *Zephyrus* hairstreaks in Asia. Map made with Natural Earth. Deep purple-coloured grid cells contain the highest number of species of *Zephyrus* hairstreaks, lower number of species are in the light purple cells; for white grid cells no species have been recorded.

Three centres of species richness of *Zephyrus* hairstreaks are situated in the eastern Qinghai-Tibet Plateau (EQTP), the Hengduan Mountain Region (HDMR), and the Qinling Mountain Region (QLMR). The highest values of the centres of species richness reached 74, 60 and 48 in one grid cell of 2°×2° in these three areas, respectively. The remaining portions of the mountains and islands also contain a relatively high abundance, such as the tri-border region of China, Laos and Vietnam (TCLV, value 29), Taiwan island, China (value 25), the Wuling Mountain Region (WLMR) located in central China and the Tianmu Shan region (TMS) (value both 24), Japan (value 23), the Russian Far East (value 20), the Korean peninsula (value 19), the Changbai Mountain region and the Nanling Mountain region (NLM) of China (value both 17), the eastern Himalaya (EHMLY, value 16), Hainan Island, China, and Sakhalin Island, Russia (values of both 6). Some species were also unevenly distributed in the chain of the Himalaya (Fig 1).

### Latitudinal gradients in species richness

**Fig 2:**
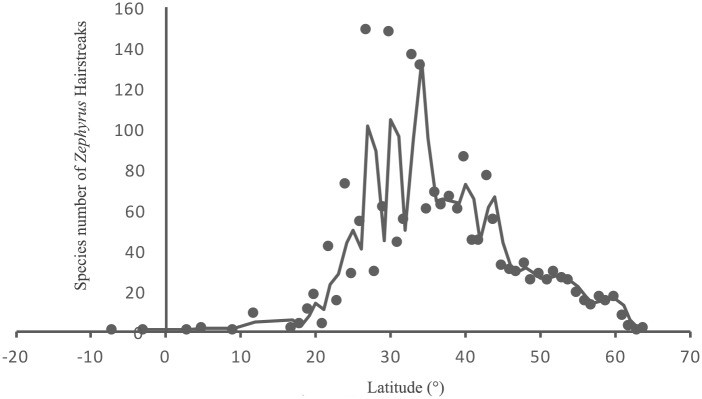
Species richness of *Zephyrus* hairstreaks by latitudinal gradient. Latitudinal gradients in species richness show a normal distribution (Kolmogorov-Smirnov Z = 1.281, P = 0.075 > 0.05) with one clear peak between 25° N and 35° N in the temperate zone, and a gradual decrease towards the poles.

The value of species richness of *Zephyrus* hairstreaks in each grid cell is variable in Eurasia. Although for certain grid cells, where the species have not been observed, this may either represent real absences or sampling errors, the database meets the analysis criterion and reflects reasonable real world results. Latitudinal gradients in species richness show a normal distribution (Kolmogorov-Smirnov Z = 1.281, P = 0.075 > 0.05) with one clear peak between 25° N and 35° N in the temperate zone (Fig 2), and a gradual decrease towards the poles. The prevalent consensus regarding species richness is that diversity generally peaks in the tropics near the equator and declines towards the poles.

### Areas of endemism

**Fig 3:**
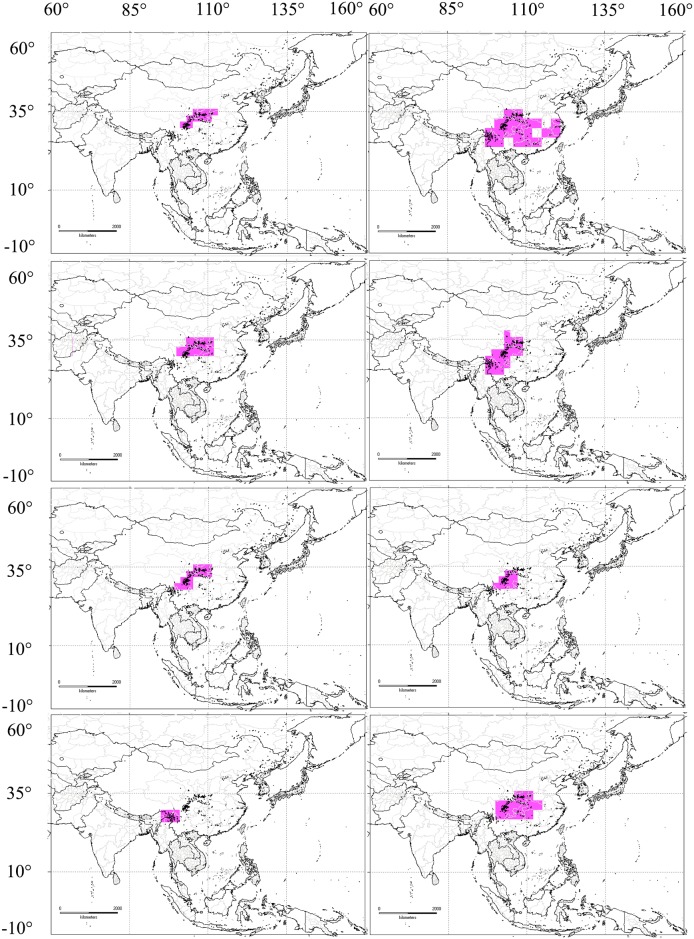
Consensus areas of endemism detected for *Zephyrus* hairstreaks by NDM/VNDM in Asia. Map made with Natural Earth. Areas of endemism at generic level: 2.0° (a) and 3.0° (b, c); Areas of endemism at specific level 2.0° (d-g) and 3.0° (h). Lower cases indicate those areas assembled over different regions: (a, c, e): QLMR-EQTP. (b): QLMR-EQTP-WLMR-TMSM-HDMR-NLM. (d, m): QLMR-EQTP-HDMR. (f): EQTP. (g): HDMR. (h): QLMR-EQTP-WLMR.

**Fig 4:**
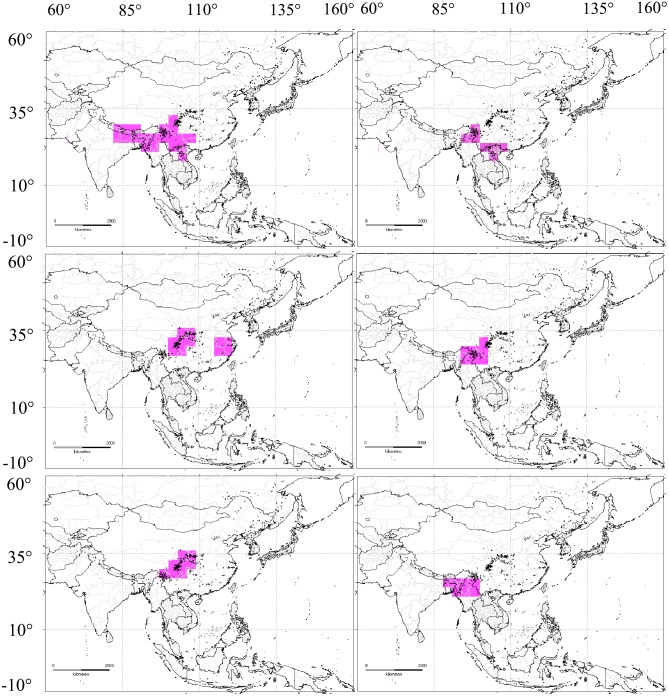
Consensus areas of endemism detected for *Zephyrus* hairstreaks by NDM/VNDM in Asia. Map made with Natural Earth. (i): EQTP-HDMR-EHMLY-TCLV. (j): HDMR-TCLV. (k): QLMR-EQTP-TMS. (l): EQTP-HDMR. (m): QLMR-EQTP-HDMR. (n): EHMLY.

The NDM/VNDM 3 analytic approach identified fourteen different AoEs (Figs 3 and 4) at two taxonomic levels (genus and species) and two different Operative Geographical Units (2.0°×2.0° and 3.0°×3.0°). At the genus level, there were three consensus endemic areas (Fig 3a–3c) searched, using the heuristic algorithm of NDM/VNDM 3 and supported by the values of 2.13–2.38 (Fig 3a in 2.0° grid size), 2.26–3.26 (Fig 3b in 3.0° grid size), and 2.26–2.51 (Fig 3c in the 3.0° grid size), respectively. The consensus endemic areas of Fig 3a and 3c were formed of the same three genera (*Neogonerilia*, *Saigusaozephyrus* and *Shaanxiana*) but in different proportions (Table in S2 Table), with both of these two regions being associated with the eastern Qinghai-Tibet Plateau and the Qinling Mountain Region (EQTP-QLMR). Areas of endemism from Fig 3b (QLMR-EQTP-WLMR-TMS-HDMR-NLM) mainly covered central and southern China. At the specific level, there were eleven overlapping or non-consensus endemic areas identified, based on two different Operative Geographical Units (2.0° and 3.0°). They were assembled over different regions; the Qinling Mountain Region (QLMR), the eastern Qinghai-Tibet Plateau (EQTP), the Wuling Mountain Region (WLMR), the Tianmu Shan region (TMS), the Hengduan Mountain Region (HDMR), the Nanling Mountain Region (NLM), the eastern Himalaya (EHMLY) and the tri-border region of China, Laos and Vietnam (TCLV). In the 2.0° grid size, there were four consensus areas (Fig 3d–3g): QLMR-EQTP-HDMR (score range 3.04–4.27), QLMR-EQTP (score range 5.71–6.46), EQTP (score range 7.64–8.14) and HDMR (score range 3.67–3.92), all of which were derived from an optimality criterion to demarcate AoEs. There were seven consensus areas (Figs 3h and 4i–4m) in 3.0° grid size; QLMR-EQTP-WLMR (score range 3.07–3.32), EQTP-HDMR-EHMLY-TCLV (score range 3.38–3.63), HDMR-TCLV (score range 3.84–4.09), QLMR-EQTP-TMS (score range 4.34–4.59), EQTP-HDMR (score range 3.97–4.22), QLMR-EQTP-HDMR (score range 17.33–17.58) and EHMLY (score range 3.30–3.55).

### The core areas of endemism

**Fig 5:**
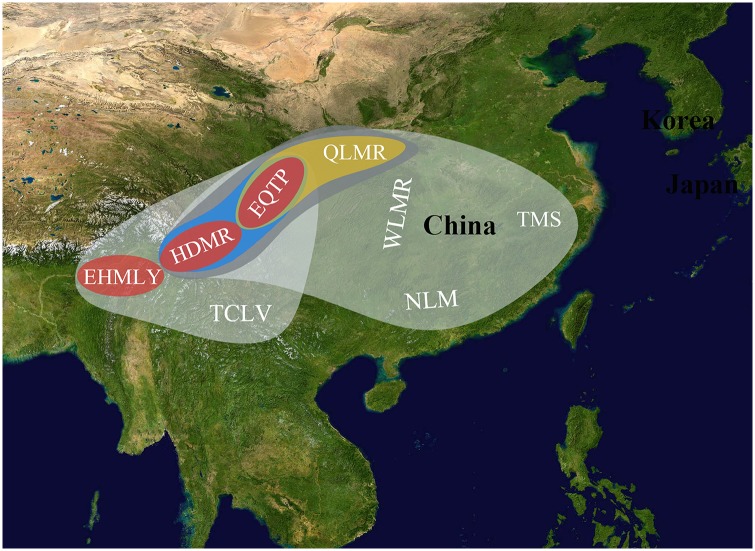
Areas of endemism and their locations. Map from Visible Earth. **QLMR**: Qinling Mountain Region. **EQTP**: Qinghai-Tibet Plateau. **HDMR**: Hengduan Mountain Region. **EHMLY**: Eastern Himalaya. **TCLV**: Tri-border region of China, Laos and Vietnam. **WLMR**: Wuling Mountain Region. **TMS**: Tianmu Shan. **NLM**: Nanling Mountain.

Three core consensus AoEs (EQTP, HDMR and EHMLY) (Fig 5) had high consensus scores of endemicity and could be identified at the specific level. EQTP is supported as an independent AoE in the 2.0° grid size by 15 species with differing consensus scores; *Chrysozephyrus linae* (0.303–0.324), *Chrysozephyrus marginatus* (0.688), *Chrysozephyrus sakula* (0.625), *Cordelia koizumii* (0.545), *Gonerilia pesthis* (0.393), *Hayashikeia courvoisieri* (0.688), *Hayashikeia florianii* (0.289), *Howarthia caelestis* (0.364–0.391), *Howarthia nigricans* (0.719), *Howarthia sakakibarai* (0.639), *Neozephyrus coruscans* (0.000–0.344), *Neozephyrus helenae* (0.719), *Teratozephyrus chibahideyukii* (0.719), *Teratozephyrus hecale* (0.463) and *Uedaozephyrus kuromon* (0.438). Compared with that, there were five species in the consensus AoE (HDMR) in the 2.0° grid size with different contribution scores; *Chrysozephyrus yunnanensis* (0.750), *Euaspa mikamii* (0.750), *Neozephyrus dubernardi* (0.833), *Shirozuozephyrus nansarae* (0.667) and *Shirozuozephyrus nyishwini* (0.667). Among the three core AoEs, EQTP and HDMR at the same time have high species richness. In the 3.0° grid size, SHEMY was also identified as a core consensus AoE, supported by five species; *Chrysozephyrus letha* (0.714), *Chrysozephyrus uedai* (0.633), *Neozephyrus suroia* (0.455), *Shirozuozephyrus jakamensis* (0.800) and *Shirozuozephyrus khasia* (0.700). Additionally, the consensus area of QLMR-EQTP-HDMR (Fig 5) was obtained from the analysis results using two grid sizes (2.0° and 3.0°) with its highest support scores (up to 17.32973–17.57973) made up of 28 species (in the 3.0° grid size) (Table in S2 Table) based on NDM/VNDM 3. QLMR-EQTP (Fig 5) was strongly supported as an AoE for the reason that, at the generic level, it was supported by three genera with different scores in the 2.0° and 3.0° grid sizes; *Neogonerilia* (0.600/0.800), *Saigusaozephyrus* (0.680/0.605) and *Shaanxiana* (0.850/0.857). Furthermore, at the species level, there were twelve species supporting an endemic area in the 2.0° grid size (Table in S1 Table); *Chrysozephyrus gaoi* (0.469–0.591), *Chrysozephyrus souleanus* (0.000–0.306), *Chrysozephyrus tatsienlurnsis* (0.444–0.455), *C*. *koizumii* (0.750–0.763), *G*. *pesthis* (0.750–0.833), *Gonerilia thespis* (0.000–0.621), *N*. *coruscans* (0.643–0.682), *Saigusaozephyrus atabyrius* (0.646–0.854), *Shaanxiana takashimai* (0.630–0.792), *Shirozua melpomene* (0.403–0.419), *T*. *hecale* (0.000–0.537) and *Wagimo sulgeri* (0.000–0.556). It is worth noting that while both EQTP and HDMR are centres of species richness and AoEs which are located adjacent to each other, they appear independent, differing with each other. For instance, both EQTP and HDMR are supported as independent AoEs in the 2.0 grid size, and moreover, EQTP-HDMR (Fig 5) has a lower support rate (only one time) and values (3.974–4.224) compared with those of QLMR-EQTP (three times and their values as follows: 2.130–2.380, 2.262–2.512 and 5.714–6.464). QLMR-EQTP-HDMR is treated as a large and important region for species richness and AoEs of *Zephyrus* hairstreaks and among these areas QLMR has a particularly closer connection with EQTP (because QLMR lacks independence and was three times recognised together with EQTP) more than other AoEs.

## Discussion

### Areas of endemism and species richness

Prado *et al*. [59] stated that using smaller grid cells appears to be more restrictive and rigorous in identifying AoEs but it may lead to ignoring some important areas, so we identified these regions using different grid sizes and different taxonomic levels. The results revealed the most regions in the mountains of central and southern China, especially the area of QLMR-EQTP-HDMR (Fig 5) as AoEs. These regions played a significant role as centres of survival and speciation during the Quaternary global cooling [4], and these AoEs (Fig 5) appear consistent with those obtained from other biological assemblages in plants, birds and aphids [63, 64]. The highest species richness value within the area of EQTP reached 74. The Hengduan Mountain Region (HDMR) also recorded 60 species of *Zephyrus* hairstreaks. The two areas are not only core AoEs but also centres of species richness. In addition, the species compositions within the two regions appear markedly different (Table in S2 Table). EHMLY (Fig 5) is also a core AoE, independent from HDMR and EQTP, although it lacks high species richness. Eliot [16] deduced the greatest abundance and variety of Lycaenidae in the SE Asian Subregion of the Oriental Region, and he suggested that the SE Asian Subregion extends from SE China to Ceylon and as far east as Weber’s Line. Its characteristic species are lowland or submontane in habit and appear to be centred in Sundaland. However, centres of species richness of *Zephyrus* hairstreaks and core AoEs are located in the Sino-Himalayan and Sino-Japanese subregions.

### General distribution and diffusion of *Zephyrus* hairstreaks

**Fig 6:**
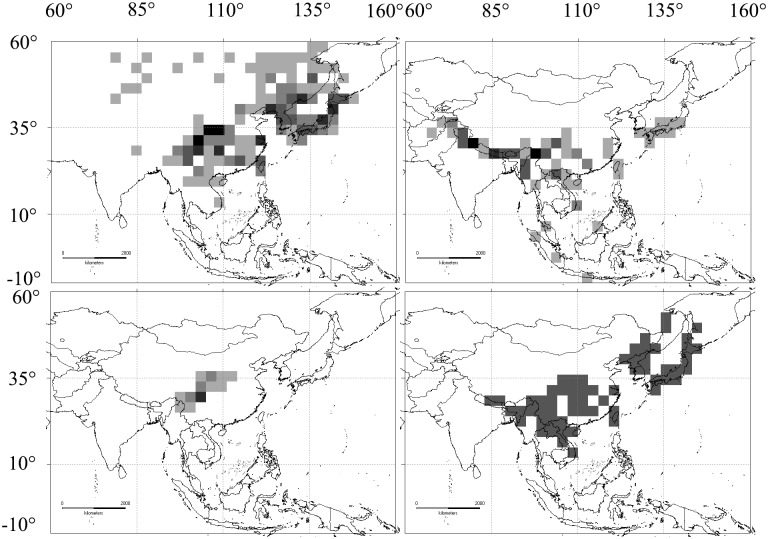
Four major distribution patterns of *Zephyrus* hairstreaks in Asia. (a): Sino-Japanese distribution. (b): Sino-Himalayan distribution. (c): High-mountain distribution. (d): Combined distribution covering all three above patterns (*Chrysozephyrus*).

Almost all of the *Zephyrus* hairstreaks are distributed in the Northern Hemisphere and a major part of them are concentrated in Asia, except for three species in North America and two species in Europe. Our data used in the analyses showed that the northern boundary of distribution of *Zephyrus* hairstreaks is located along the Sea of Okhotsk in the Russian Far East in Asia, with the southern boundary located in Java in Indonesia, the western boundary is in the eastern region of Afghanistan, and the eastern boundary is in northern Japan and its surroundings, In addition, parts of northern and northwestern China are covered by large areas of desert and inaccessible areas, so there are no distributional records of *Zephyrus* hairstreaks in our database, along with areas of over-urbanization and areas incompletely investigated, all of which are represented as gaps (Fig 1). Nevertheless, our database maximizes the use of published available information and investigations of our team and fulfills the criteria of quality of biodiversity databases discussed by Hortal et al., [65]. At last, there are four major distribution patterns of *Zephyrus* hairstreaks in Eurasia (Fig 6): 1) Sino-Japanese distribution: the species belonging to this distribution pattern are characteristic with extensions from EQTP and HDMR northward to Manchuria-Pacific (Northeast China, the Korean Peninsula, the Russian Far East and Japan), southward to South China (WLMR, TMS and NLM), and even to the west of Europe (e.g. *Neozephyrus*, *Shirozua*, *Iozephyrus*, *Thecla* and *Howarthia*). 2) Sino-Himalayan distribution: the species originated on the EQTP and HDMR, from where it dispersed to northwestern India along the Himalayan chain and eastward to east China, Indochina, Sundaland, Taiwan island, Japan and the west of Wallacea (e.g. *Euaspa*, *Shirozuozephyrus*, *Chaetoprocta* and *Austrozephyrus*). 3) High mountain distribution: the species are distributed only in the areas of HDMR, EQTP and QLMR (e.g. *Kameiozephyrus*, *Saigusaozephyrus* and *Uedaozephyrus*). 4) Combined distribution: the species exhibit a combined distribution pattern covering all three other patterns (*Chrysozephyrus*).

Evidence suggests that the Indo-Asian collision occurred in the early Cenozoic Era, 50–55 Myr ago or even earlier [66], and it almost simultaneously caused the uplift of the Himalaya-Tibetan plateau region and the Eurasian plate, which also underwent glacial/interglacial cycles during the Quaternary [4]. Lepidoptera, as the second largest order of Insecta, diverged prior to the Cretaceous/Paleogene (K/Pg) event (65 Mya) and the extant families of Lycaenidae quite likely evolved in the Cenozoic Era [67]. In addition, the extrusion of the Indochina block [68–70] which happened during the period of 23 to 24 Mya [71] was proposed to explain the formation of the boundary line called the “Tanaka-Kaiyong line” which extends from 28° N, 98° E southward to approximately 18° 45′or 19° N, 108° E [13, 14, 72, 73]. Of particular note, this biogeographical line, to some extent, exists between the core AoEs of EQTP and HDMR. This fact indicates that *Zephyrus* hairstreaks may have originated at least 23–24 Mya, and the Cenozoic orogeny as a result of the Indo-Asian collision likely caused rapid speciation in two AoEs, EQTP and HDMR. The significant differences between EQTP and HDMR (identified as independent AoEs respectively with lower correlation values) probably can be attributed to vicariance events which happened in these areas, for instance, the aforementioned extrusion of the Indochina block.

## Conclusion

Areas of endemism and centres of species richness of *Zephyrus* hairstreaks are located in central and southern China, the Himalaya as well as northern Indo-China which is in good agreement with Quaternary vegetation reconstructions [4, 74] that had been supposed as the existence of refugia in China. Furthermore, four distribution patterns inferred two dispersal routes of endemism and diversity, which strongly suggests that *Zephyrus* hairstreaks probably originated in E. Asia between 25°N to 35°N in the temperate zone (based on our database). Dispersal may have played a leading role in the closer relationship of species between QLMR and EQTP, while vicariance events are the most likely cause of the significant differences between EQTP and HDMR. In addition, the Cenozoic orogeny (the uplift of the Himalaya-Tibetan plateau region and the extrusion of the Indochina block during the Indo-Asian collision) [66, 71], probably accelerated speciation, especially in the core areas endemism.

## Supporting information

S1 Table Distributional data of *Zephyrus* hairstreaks. (XLS)

S2 Table The values of areas of endemism. (XLS)
